# Using iron sucrose-labeled adipose-derived mesenchymal stem cells in 1.5 and 3 T MRI tracking: An *in vitro* study

**DOI:** 10.1016/j.heliyon.2020.e04582

**Published:** 2020-08-01

**Authors:** Paween Tangchitphisut, Narongrit Srikaew, Sith Phongkitkarun, Suphaneewan Jaovisidha, Tulyapruek Tawonsawatruk

**Affiliations:** a Department of Orthopaedics, School of Medicine, Mae Fah Luang University, Thailand; b Research and Innovative Center, Ramathibodi Hospital, Thailand; c Department of Radiology, Faculty of Medicine, Ramathibodi Hospital, Thailand; d Department of Orthopaedics, Faculty of Medicine, Ramathibodi Hospital, Thailand

**Keywords:** Bioengineering, Tissue engineering, Stem cells research, Regenerative medicine, Orthopedics, Radiology, Iron sucrose, Tracking, Tracer, Mesenchymal stem cells, MRI

## Abstract

**Objectives:**

The objective of this study was to investigate iron sucrose labeling in mesenchymal stem cell (MSCs) tracking.

**Background:**

Adipose-derived mesenchymal stem cell-based therapy is a promising strategy for promoting musculoskeletal repair.

**Methods:**

Iron sucrose-labeled adipose-derived mesenchymal stem cells (IS-labeled ASCs) were tracked using T2-and T2∗-weighted sequences by 1.5 and 3 T MRI in an *in vitro* model. ASCs were isolated from cosmetic liposuction specimens. ASCs from passages 4–6 were labeled with iron sucrose (Venofer®) which was added to the cell culture medium. Pre- and post-iron sucrose labeled ASCs were evaluated for cell surface immunophenotypes. Cell viability as well as chondrogenic, adipogenic and osteogenic differentiation of IS-labeled-ASCs were evaluated. The IS-labeled ASCs were titrated into microtubes at 1 × 10^3^, 1 × 10^4^, 1 × 10^5^ and 1 × 10^6^ cells/ml/microtube and their intensities were determined by 1.5 and 3T MRI using T2-and T2∗-weighted sequences.

**Results:**

The expression markers of IS-labeled ASCs from flow cytometry were equivalent to control. The mean cell viability was 97.73 ± 2.06%. Cell differentiations of IS-labeled ASCs were confirmed in each lineage using specific staining solutions. T2∗-weighted sequences (T2∗) were able to detect iron sucrose labeled-ASCs at a minimum of 1 × 10^5^ cells/ml/microtube using 1.5 and 3T MRI, but the detection sensitivity was lower with T2-weighted sequences (T2).

**Conclusions:**

Iron sucrose incubation is a safe alternative method for ASCs labeling and tracking using MRI following treatment. Clinicians and researchers should be able to visualize the location of ASCs engraftment without secondary surgical investigation involving tissue sampling.

## 1 Introduction

Presently, mesenchymal stem cells (MSCs) are widely used both in orthopaedic research and in clinical practice as part of intra-articular injection and bone/cartilage scaffold implantation [1, 2, 3, 4, 5]. Treatment with MSCs in animal and human studies has shown improvement in cartilage healing including reduction in the size of cartilage defects, reduced pain scores, increased quality of life scores (e.g., WOMAC score, KKOS score), improved radiographic findings and reduced synovial inflammation [6, 7, 8, 9, 10]. As well as for treatment of cartilage defects, MSCs could also be used in the treatment of bone defects e.g., non-union fracture, tendon injury, meniscus injury and intervertebral disc degeneration with or without scaffold implantation [11, 38].

The mechanisms of cartilage and intra-articular healing after MSCs injection, however, are not clearly understood. MSCs might act as immunomodulators by releasing growth factors and anti-inflammatory cytokines resulting in an optimal intra-articular environment for tissue healing and promoting tissue healing [12, 13, 14]. Additionally, MSCs might be becoming engrafted at the site of the tissue defects where they promote local tissue repair [13]. Clarification of the mechanisms of MSCs engraftment requires direct visualization by secondary invasive intervention, e.g., arthroscopic examination, tissue biopsy or tissue excision. However, ethical issues must be considered in cases where the patient does not meet the criteria for surgery. This cell tracking method provides a potential new method for indirect visualization of MSCs in a patient's joint or other organs following MSCs injection or implantation.

One of the cell tracers currently used for observing localization, cell migration and cell function is superparamagnetic iron oxide (SPIOs) [15]. SPIOs have been approved by the U.S. FDA as safe for use in humans, but their high cost means they are not available in many countries and markets [16]. Iron sucrose (Venofer®, Vifor (International) Inc., Switzerland.) has been suggested as a new type of MRI cell tracer which has not yet been used in either research or clinical practice. It is one of the drugs currently used for treatment of iron-deficiency anemia and anemia associated with chronic kidney disease [17]. Properties of Venofer® include being a hypertonic (1,250 mOsmol/L) and base pH (10.5–11.1) solution [18]. The structure of Venofer® consists of iron (III)-hydroxide cores surrounded by many non-covalently bound sucrose molecules [19]. This complex molecule is similar in structure to ferritin. Most human cells have transferrin receptor (CD-71) that uptakes transferrin-iron complexes to the cells by the process of receptor-mediated endocytosis [20]. After that, ferritin and ferritin-like complex is stored in cytosol and mitochondria [21]. *In vitro* studies have shown that iron sucrose does not adversely affect either human bone marrow-derived mesenchymal stem cell viability or chondrogenic differentiation [22].

The authors are aware of no studies which have investigated and evaluated iron sucrose-labeled mesenchymal stem cells (IS-labeled MSCs) using magnetic resonance imaging (MRI). MRI is a non-invasive technique which can be used for detecting iron deposition and iron concentration in many organs, e.g., heart, liver, pancreas and pituitary gland [23, 24]. This study hypothesized that magnetic resonance imaging (MRI) might be able to detect IS-labeled MSCs.

## 2 Objectives

This study aimed to demonstrate the indirect visualization of IS-labeled ASCs using 1.5 and 3T MRI in an *in vitro* model. A secondary objective was to determine the most sensitive technique for detection and identification of the point of cell concentration of IS-labeled ASCs detectable using MRI.

## 3 Methods

### 3.1 Study design and patients

This *in vitro* experimental study was conducted from April 2016 to October 2017 at Ramathibodi Hospital, Bangkok, Thailand. The study was one of series of studies on the theme “Efficacy of Autologous Adipose Mesenchymal Stem Cells Versus Hyaluronic Acid Intra-articular Injection in Knee Osteoarthritis: Phase 1 and 2” and was approved by the Committee on Human Rights Related to Research Involving Human Subjects, Faculty of Medicine, Ramathibodi Hospital, Thailand (MURA2014/476 and MURA2015/12). This study was exempted from the requirement to acquire patient consent for collecting waste specimens from routine surgery. Informed consent for the routine surgeries was obtained from the patients prior to their operation. Waste subcutaneous adipose tissue samples were obtained from 5 anonymous female patients (average age 30.40 ± 13.16 years) who had undergone elective cosmetic liposuction. ASCs were extracted from all samples, then held in the liquid nitrogen storage cell bank at the Research Center, Ramathibodi hospital.

### 3.2 ASCs culture technique and immunophenotype assessment

The cell culture technique used in this study followed the method described in Tangchitphisut et al. [25]. ASCs were defrosted in a warm water bath for about 1 min then reactivated with 5 ml of Dulbecco's Modified Eagle Medium-low glucose (DMEM-LG) (Biochrom®, Berlin, Germany) with 10% fetal bovine serum (FBS) (Biochrom®, Berlin, Germany) and centrifuged at 3,000 g for 3 min. The surfactant layer was then removed. The retained pellet was mixed with 10 ml of phosphate buffer solution (PBS), then passed through a sterile filter (Corning® cell stainer, NY, USA) and centrifuged at the same speed. The cell pellet was re-suspended in 1 ml of complete medium (DMEM-LG + 10% FBS + 1% L-glutamic acid (Gibco®, NY, USA) + 1% Penstrep (Gibco®) + 0.1% Amphotericin-B (Fungizone®, NY, USA) then divided for cell counting using a hemocytometer. Cells were subsequently seeded in T-25 tissue culture flasks at a density of 5,000 cell/cm^2^ at 37 °C (humidified atmosphere 95% O_2_ and 5% CO_2_). The medium was changed every 2 days. On days 7–10, the cells were examined and microscopic appearance observed. If cells covered more than 80% of the culture flask, ASCs were detached with 0.05% Trypsin/0.1% EDTA (Gibco®, Canada) and re-cultured as the first passage with a complete medium through the 4^th^ to 6^th^ passage (about 1 × 10^8^ cells). ASCs in passages 4–6 were trypsinized and divided into a control group and an iron sucrose (Venofer®) transfected group.

### 3.3 Iron sucrose transfection and labeling technique

One mg/ml (complete medium) of iron sucrose (Venofer®) was added to the basal culture medium then the mixture was re-incubated at 37 °C for 16 h [17]. The dose was based on that used in previous studies [22, 26] After that, the transfected medium was washed out 3 times using PBS and then trypsinized and was then split for:

1. Cell viability evaluation using the Trypan-blue exclusion method [27] using a hemocytometer and a light microscope.
2. Immunophenotypes assessment to confirm MSCs characteristics with flow cytometry using positive cell surface markers (CD73 and CD90) and negative cell surface markers (CD 34 and CD45) (eBioscience, USA).
3. Determination of IS-labeled ASCs' adipogenic-, osteogenic-, and chondrogenic differentiation potential.
4. Histopathology assessment of IS-labeled ASCs with Prussian blue staining [37] under a light microscope (Nikon® eclipse TE2000-U).
5. Preparation of IS-labeled ASCs for MRI assessment.

### 3.4 Cell differentiation potential of IS-labeled ASCs

The cell differentiation technique used in this study was proposed in a previous paper [20]. Chondrogenic, adipogenic and osteogenic differentiation potentials were determined. 5 × 10^6^ cells from the 4^th^ to the 6^th^ passages of IS-labeled ASCs were split and cultured in specific medium T-25 flasks for adipogenic and osteogenic differentiation, but were cultured in 6 well plates for chondrogenic differentiation and for cartilage formation. The period of ASCs culture in the specific medium was 21 days.

Cartilage (cartilage pellet) formation was cultured as a two-dimensional (2D) chondrogenic differentiation model. This study used a commercial medium from PromoCell®, USA. Adipogenic and osteogenic differentiation mediums were combined with Dulbecco's Modified Eagle Medium-low glucose (DMEM-LG), Ham's-12, 10% FBS, 1% L-glutamic acid and 1% Penstrep. Adjunction of isobutyl methylxanthine (IBMX) (Sigma®, Japan), Dexamethasone (Sigma®, Japan), Insulin (Sigma®, Japan) and Indomethacin (Sigma®, China) with the base medium and used as the adipogenic differentiation medium. Dexamethasone, β-glycerophosphate (Sigma®, USA) and Ascorbic acid (Sigma®, Japan) were combined with a base medium to make up the osteogenic differentiation medium.

On day 21, the cells were stained with specific staining to identify the cells’ histology. Chondrogenic-induced ASCs were stained with Alcian blue staining solution (stains dark blue for aggrecan) [38]. Osteogenic-induced ASCs were stained with Alizarin red staining solution that detects extracellular calcium deposition (bright orange-red) [38]. Adipogenic-induced ASCs were stained with Oil Red-O (intracellular lipid vesicles) (bright red) [38].

### 3.5 Preparation of IS-labeled ASCs for MRI assessment

**Image 1:**
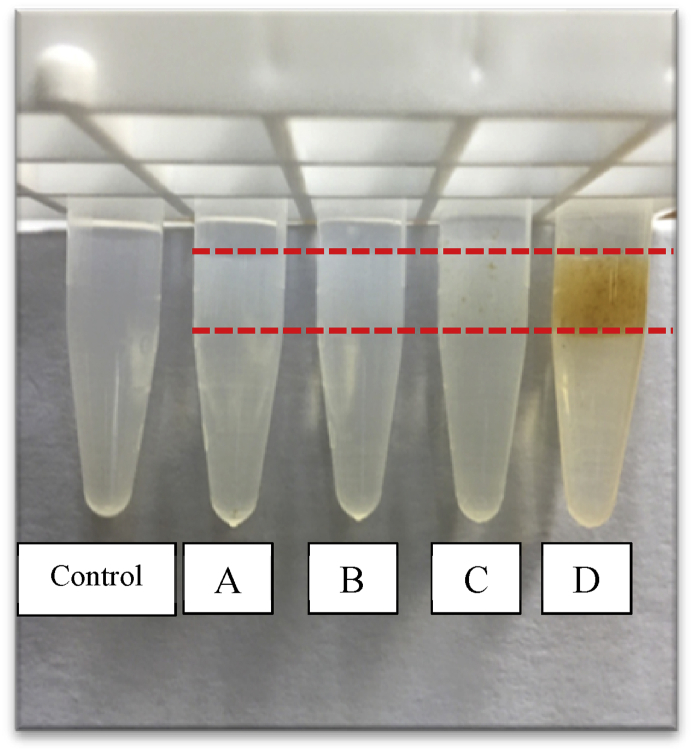
IS-labeled ASCs in microtubes (between dash lines). A = 1 × 10^3^ cells/ml, B = 1 × 10^4^ cells/ml, C = 1 × 10^5^ cells/ml, D = 1 × 10^6^ cells/ml.

IS-labeled ASCs cultures were titrated into 5 microtubes: control (pure agarose without IS-labeled ASCs) and 1 × 10^3^, 1 × 10^4^, 1 × 10^5^ and 1 × 10^6^ cells/ml/microtube. Preparation steps included: 1) low electroendosmosis (EEO) agarose, molecular biology grade (Research Organic®) placed as a base layer in each microtube, 2) 1 ml of titrated IS-labeled ASCs culture inserted as the middle layer, and 3) a top layer closed with low EEO agarose (Image 1). After that, the IS-labeled ASCs tubes and the control tube were inserted into a container.

### 3.6 1.5 and 3T MRI preparation and T2/T2∗ measurement

IS-labeled ASCs tubes in containers (one patient per container) were sent to the Advanced Diagnostic Imaging Center (AIMC), Ramathibodi Hospital for evaluation using a 1.5T MRI scanner (GE SIGNA EXCITE HDxt, Milwaukee, WI, USA) with an 8 ch HD TR Knee coil and a 3T MRI Scanner (Ingenia, Philips Healthcare, Best, The Netherlands) with a 16 ch Knee coil within 24 h after cell trypsinization. Three views, axial, sagittal, and coronal, were processed and T2 and T2∗ values were assessed at the center-center area of the cell layer in each view (software: T2 Map Algorithm, Functool 4.5.3, Advantage Windows, GE V.4.4_04 for the 1.5T MRI scanner and Relaxation Maps Tool V.2.1.1 in the Philips Pride Programming environment under an IDL 6.3 for the 3T MRI scanner). T2 and T2∗ values were measured independently by two senior MRI technicians using the same technique.

The settings of the 1.5T MRI for T2 assessment were TR 1000 ms, TE 6.1 ms, delta TE 6.1 8 echo, flip angle 90°, matrix 192 × 192, and slice thickness 3 mm. Parameter settings for T2∗ assessment were TR 13.8 ms, TE 1.9 ms, delta TE 3.2 ms, 16 echo, flip angle 20°, matrix 160 × 160, field of view 160 × 160 mm, and slice thickness 3 mm.

Settings of the 3T MRI for T2 assessment were TR 2000 ms, TE 13 ms, delta TE 13 ms 6 echo, flip angle 90°, matrix 160 × 160, slice thickness 3 mm. Settings of the T2∗ assessment were TR 23 ms, TE 2 ms, delta TE 1.3 ms 16 echo, flip angle 20°, matrix 160 × 160, and slice thickness 3 mm.

### 3.7 Statistical analysis

Four concentrations of IS-labeled ASCs and one control were analyzed by SPPS version 15.0 (IBM, Texas, USA). A P-value < 0.05 was considered statistically significant in this study. Analysis of variance was conducted to compare pre- and post-iron sucrose transfected ASCs immunophenotypes. Multi-level mixed effect linear regression was used to identify any significant change in T2 and T2∗ values from the control tube in each view and for each MRI scanner. A significant result for clinical application was defined as a statistically significant change of both T2 and T2∗ values in all MRI views (axial, sagittal and coronal) with the same cell concentration or the same microtube.

## 4 Results

### 4.1 Phenotype, differentiation potentials and viability of iron sucrose-labeled ASCs

**Image 2:**
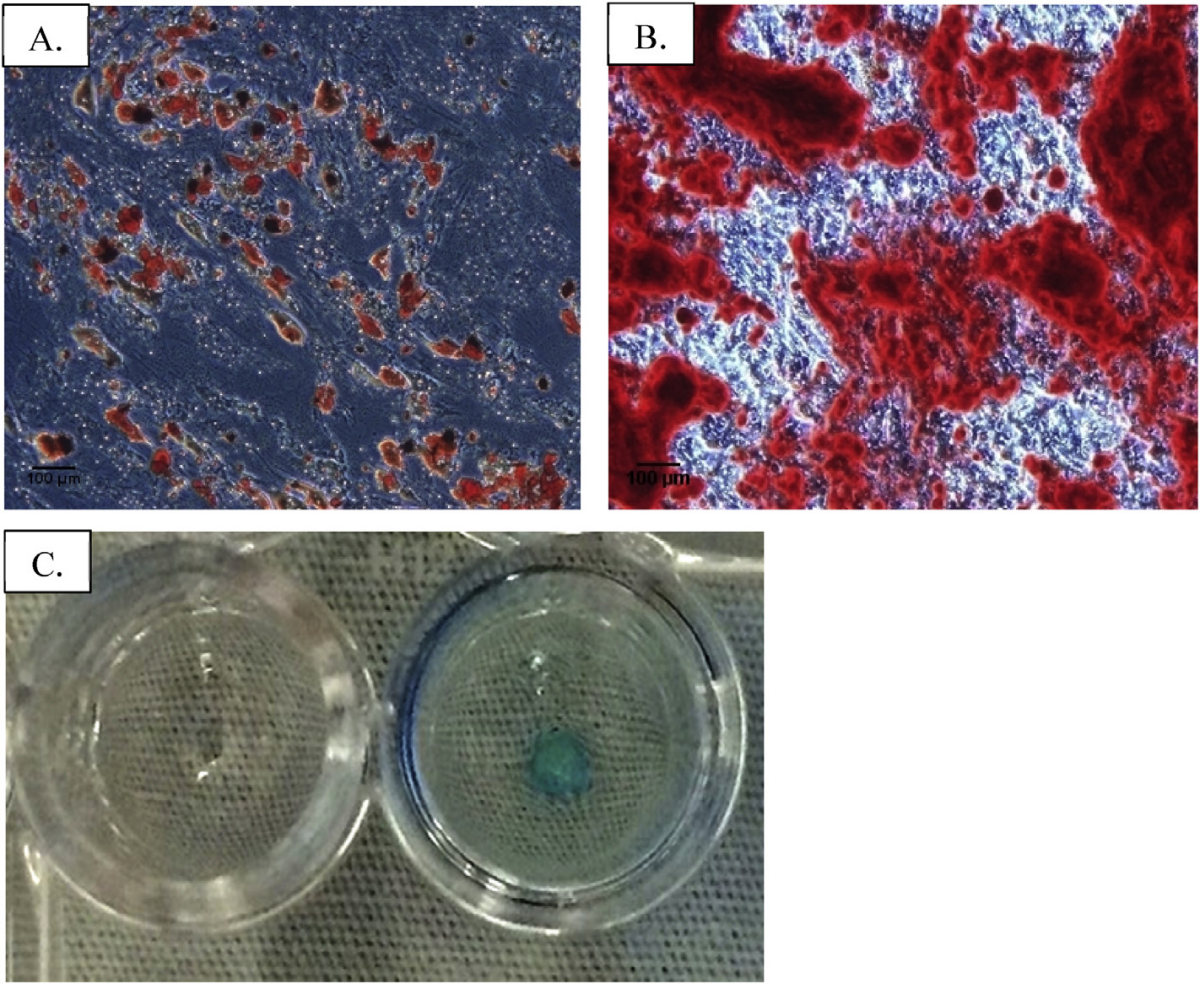
IS-labeled ASCs differentiated into (A) adipocyte (Oil Red O staining), (B) osteocyte (Alizalin red staining) and (C) cartilage pellet (Left = no staining, Right = Alcian blue staining).

Flow cytometry identified MSCs phenotypes in pre- and post-transfection ASCs. No difference in phenotypes between pre- and post-transfection ASCs was observed (Table 1). IS-labeled ASCs was still differentiated into adipocyte, osteocyte and cartilage formation (chondrocyte) as shown in Image 2. The cell viability of ASCs after iron sucrose transfection was 97.73 ± 2.06%.

**Table 1:** Comparison of ASCs’ flow cytometry pre- and post-iron sucrose transfection.

	Pre-transfection	Post-transfection	P-value
**Positive marker (%)**			

CD 73	99.4 ± 0.1	99.7 ± 0.2	0.07
CD 90	93.8 ± 8.7	98.6 ± 0.4	0.43

**Negative marker (%)**			

CD 34	0.2 ± 0.2	0.1 ± 0.0	0.39
CD 45	0.3 ± 0.1	0.2 ± 0.1	0.28

Student's t-test.

**Image 3:**
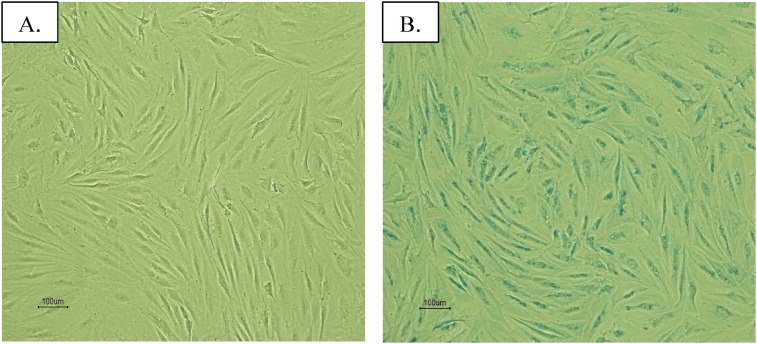
4^th^ passage of IS-labeled ASCs with a 10x light microscope. (A) Control without stain; (B) Positive blue color from Prussian blue staining.

The 4^th^ passage of IS-labeled ASCs was stained with Prussian blue staining. Image 3 shows intra-cellular blue staining in the iron sucrose labeled group.

### 4.2 IS-labeled ASCs were detected with 1.5 T MRI in T2∗-weighted sequences

**Image 4:**
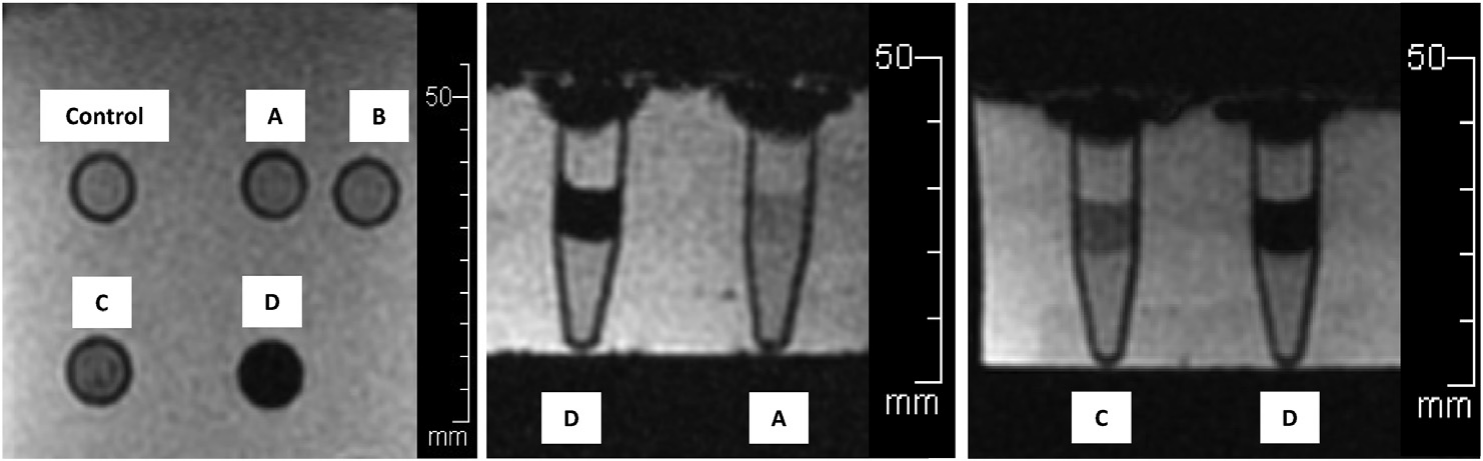
IS-labeled ASCs from T2∗-weighted sequences of 1.5T MRI. **Left**: Axial view, **Middle**: Sagittal view, **Right**: Coronal view. **(A)** 1 × 10^3^ cells/ml/microtube, **(B)** 1 × 10^4^ cells/ml/microtube, **(C)** 1 × 10^5^ cells/ml/microtube, **(D)** 1 × 10^6^ cells/ml/microtube.

**Graph 1:**
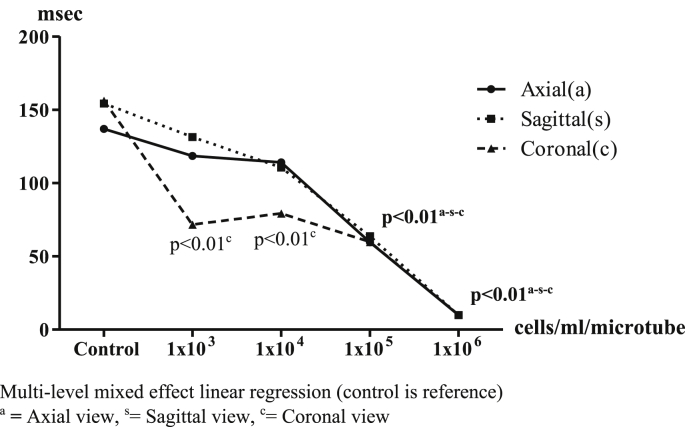
T2∗ values with 1.5 T MRI in axial, sagittal and coronal view. Multi-level mixed effect linear regression (control is reference). ^a^**=** Axial view, ^s^ = Sagittal view, ^c^ = Coronal view.

The 1.5 T MRI results showed values of signal and hypo-intensity areas in T2∗-weighted sequences could be measured as shown in Image 4. The value of most of the T2∗ signals in each view decreased inversely with the number of IS-labeled ASCs when compared with control. In axial view, the T2∗ signal values were 136.95 ± 27.33 (reference), 118.45 ± 48.16 (p = 0.36), 114.12 ± 49.22 (p = 0.26), 59.51 ± 30.62 (p < 0.01) and 9.83 ± 4.54 (p < 0.01) msec, respectively. In sagittal view, the T2∗ signal values were 154.24 ± 32.02 (reference), 131.39 ± 71.18 (p = 0.37), 110.49 ± 51.18 (p = 0.08), 63.63 ± 35.74 (p < 0.01) and 9.88 ± 4.20 (p < 0.01) msec, respectively. In coronal view, the T2∗ signal values were 156.14 ± 21.27 (reference), 71.51 ± 26.27 (p < 0.01), 79.13 ± 36.72 (p < 0.01), 59.74 ± 32.34 (p < 0.01) and 10.00 ± 4.01 (p < 0.01) msec, respectively. The 1 × 10^5^ cells/ml/microtube of IS-labeled ASCs was a significant detection point at the same time in the 1.5T MRI in T2∗ -weighted sequences (Graph 1).

### 4.3 IS-labeled ASCs were detected with 1.5 T MRI in T2-weighted sequences

**Image 5:**
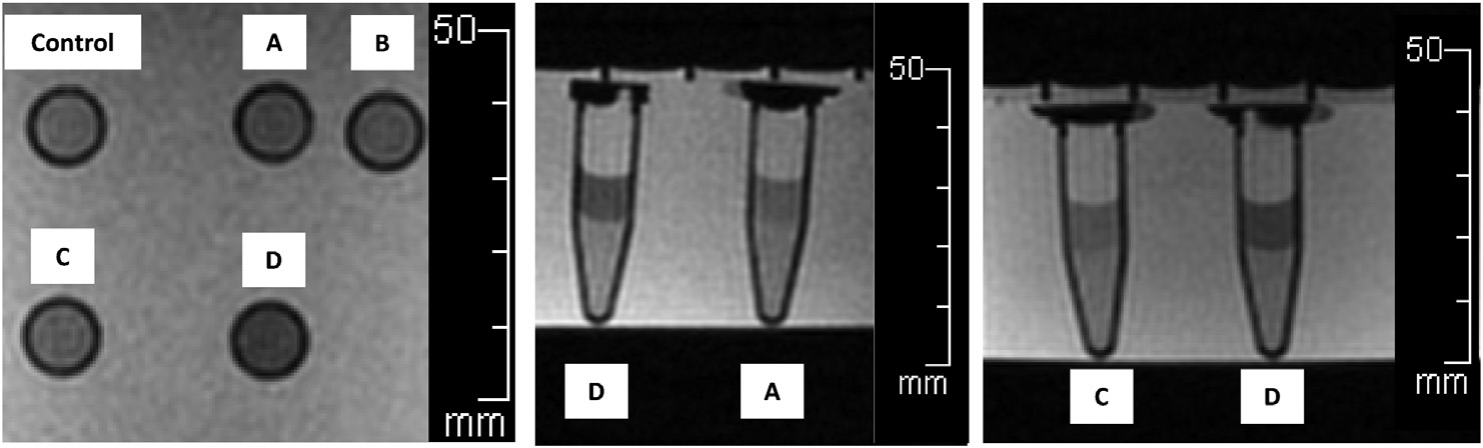
IS-labeled ASCs from T2-weighted sequences of 1.5T MRI. **Left**: Axial view, **Middle**: Sagittal view, **Right**: Coronal view. **(A)** 1 × 10^3^ cells/ml/microtube, **(B)** 1 × 10^4^ cells/ml/microtube, **(C)** 1 × 10^5^ cells/ml/microtube, **(D)** 1 × 10^6^ cells/ml/microtube.

**Graph 2:**
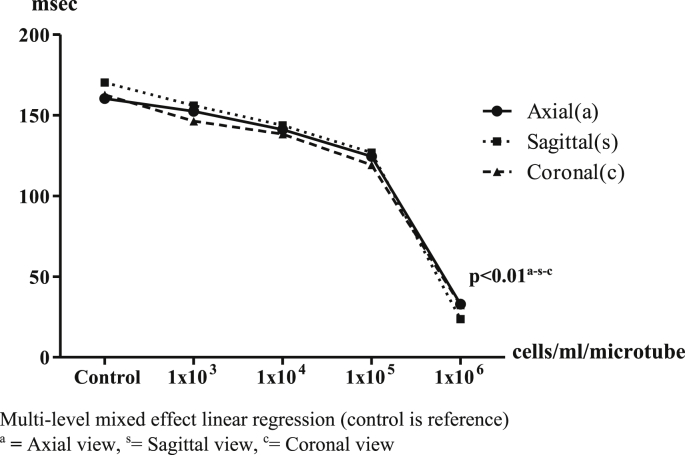
T2 values from 1.5 T MRI in axial, sagittal and coronal view. Multi-level mixed effect linear regression (control is reference). ^a^**=** Axial view, ^s^= Sagittal view, ^c^= Coronal view

The results of the 1.5T MRI showed it could measure values of signal and hypo-intensity areas in T2-weighted sequences (Image 5). Most of the T2 signal values in each view showed inversely decreasing numbers depending on the number of IS-labeled ASCs compared with control. In axial view, the T2 signal values were 160.33 ± 25.36 (reference), 152.53 ± 71.42 (p = 0.77), 141.22 ± 62.37 (p = 0.48), 124.51 ± 39.53 (p = 0.18) and 32.89 ± 6.88 (p < 0.01) msec, respectively. In sagittal view, the T2 signal values were 170.41 ± 38.57 (reference), 156.08 ± 84.90 (p = 0.67), 143.71 ± 72.48 (p = 0.43), 126.90 ± 59.29 (p = 0.19) and 23.60 ± 6.48 (p < 0.01) msec, respectively. In coronal view, the T2 signal values were 162.72 ± 30.34 (reference), 146.34 ± 72.63 (p = 0.57), 138.32 ± 65.19 (p = 0.40), 119.21 ± 52.70 (p = 0.14) and 32.42 ± 5.10 (p < 0.01) msec, respectively. The 1 × 10^6^ cells/ml/microtube of IS-labeled ASCs was a significant detection point at the same time in the 1.5T MRI in the T2 -weighted sequences (Graph 2).

### 4.4 IS-labeled ASCs were detected with 3 T MRI in T2∗-weighted sequences

**Image 6:**
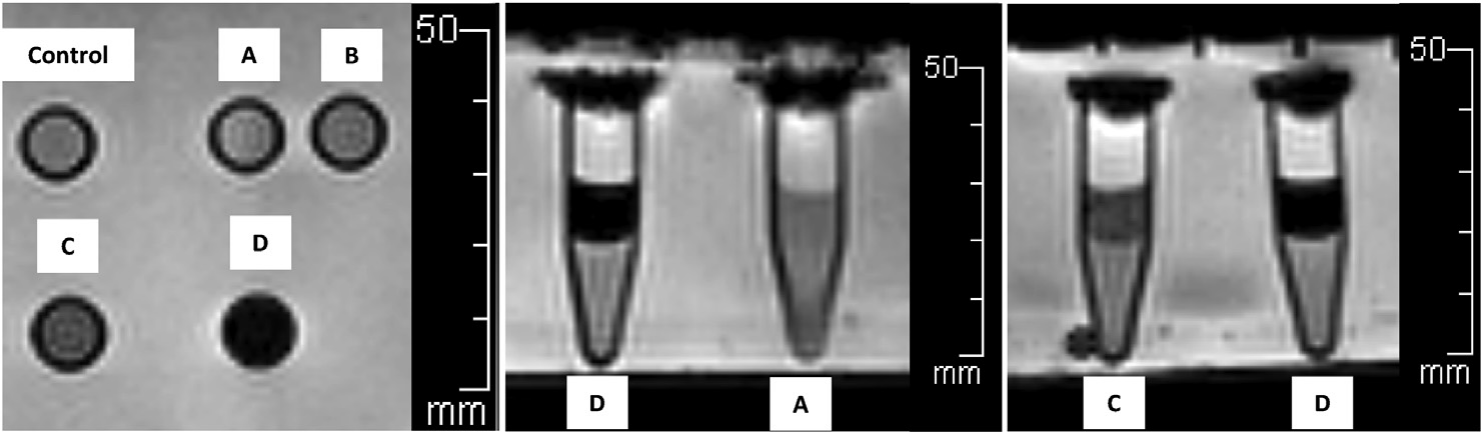
IS-labeled ASCs from T2∗-weighted sequences of 3T MRI. **Left**: Axial view, **Middle**: Sagittal view**, Right**: Coronal view. **A:** 1 × 10^3^ cells/ml/microtube, **B:** 1 × 10^4^ cells/ml/microtube, **C:** 1 × 10^5^ cells/ml/microtube, **D:** 1 × 10^6^ cells/ml/microtube.

**Graph 3:**
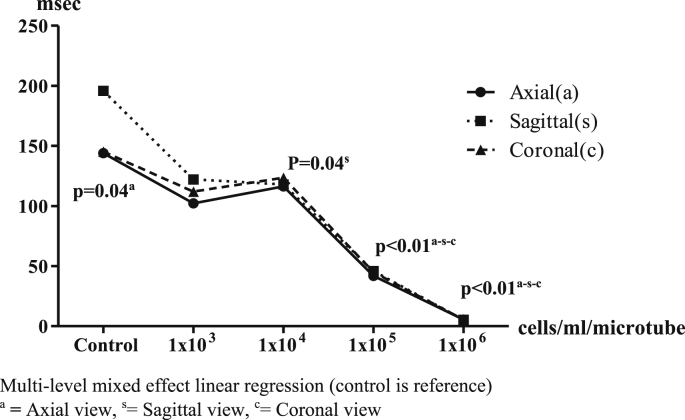
T2∗ values from 3Tesla MRI in axial, sagittal and coronal view. Multi-level mixed effect linear regression (control is reference). ^a^**=** Axial view, ^s^ = Sagittal view, ^c^ = Coronal view.

The 3 T MRI was able to measure values of signal and hypo-intensity areas in T2∗-weighted sequences (Image 6). Most of the T2∗ signal values in each view showed values inversely decreasing with the number of IS-labeled ASCs compared with control. In axial view, T2∗ signal values were 136.95 ± 27.33 (reference), 118.45 ± 48.16 (p = 0.04), 114.12 ± 49.22 (p = 0.18), 59.51 ± 30.62 (p < 0.01) and 9.83 ± 4.54 (p < 0.01) msec, respectively. In sagittal view, T2∗ signal values were 154.24 ± 32.02 (reference), 131.39 ± 71.18 (p = 0.05), 110.49 ± 51.18 (p = 0.04), 63.63 ± 35.74 (p < 0.01) and 9.88 ± 4.20 (p < 0.01) msec, respectively. In coronal view, T2∗ signal values were 156.14 ± 21.27 (reference), 71.51 ± 26.27 (p = 0.07), 79.13 ± 36.72 (p = 0.24), 59.74 ± 32.34 (p < 0.01) and 10.00 ± 4.01 (p < 0.01) msec, respectively. The 1 × 10^5^ cells/ml of IS-labeled ASCs was again a significant detection point at the same time as the 1.5T MRI in T2∗-weighted sequences (Graph 3).

### 4.5 IS-labeled ASCs were detected with 3 T MRI in T2-weighted sequences

**Image 7:**
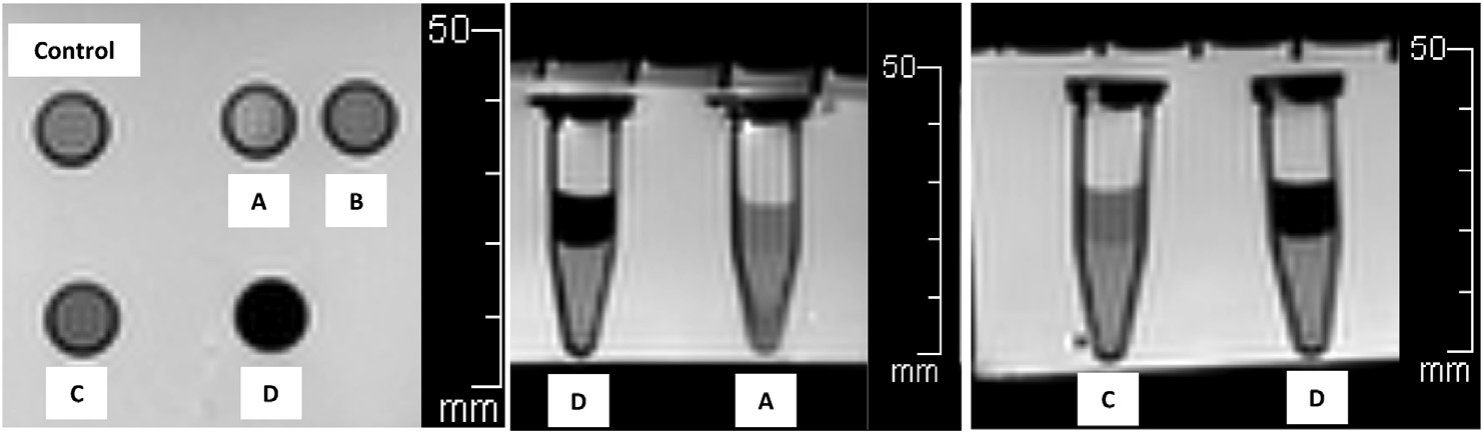
Axial view of IS-labeled ASCs from T2-weighted sequences of 3T MRI. **Left**: Axial view**, Middle**: Sagittal view**, Right**: Coronal view. **A:** 1 × 10^3^ cells/ml/microtube, **B:** 1 × 10^4^ cells/ml/microtube, **C:** 1 × 10^5^ cells/ml/microtube, **D:** 1 × 10^6^ cells/ml/microtube.

**Graph 4:**
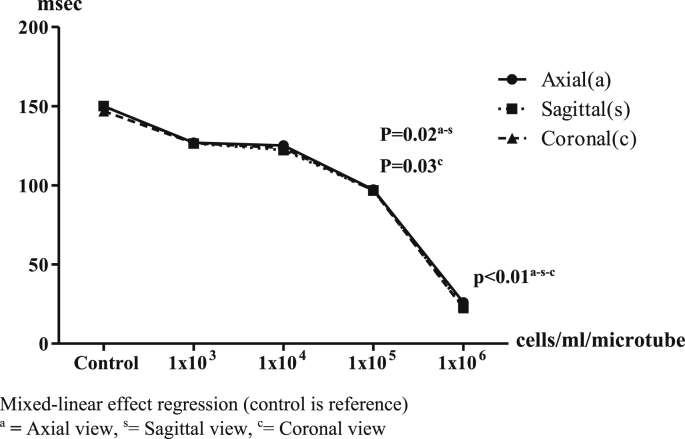
T2 values with 3 T MRI in axial, sagittal and coronal view. Mixed-linear effect regression (control is reference). ^a^**=** Axial view, ^s^ = Sagittal view, ^c^ = Coronal view.

The 3 T MRI measured values of signal and hypo-intensity areas in the T2-weighted sequences are shown in Image 7. Most of the T2 signal values in each view showed inversely decreasing values, again depending on the number of IS-labeled ASCs, compared with control. In axial view, T2∗ signal values were 136.95 ± 27.33 (reference), 118.45 ± 48.16 (p = 0.32), 114.12 ± 49.22 (p = 0.28), 59.51 ± 30.62 (p = 0.02) and 9.83 ± 4.54 (p < 0.01) msec, respectively. In sagittal view, T2∗ signal values were 154.24 ± 32.02 (reference), 131.39 ± 71.18 (p = 0.31), 110.49 ± 51.18 (p = 0.23), 63.63 ± 35.74 (p = 0.02) and 9.88 ± 4.20 (p < 0.01) msec, respectively. In coronal view, T2∗ signal values were 156.14 ± 21.27 (reference), 71.51 ± 26.27 (p = 0.37), 79.13 ± 36.72 (p = 0.30), 59.74 ± 32.34 (p = 0.03) and 10.00 ± 4.01 (p < 0.01) msec, respectively. The 1 × 10^6^ cells/ml/microtube of IS-labeled ASCs was again the detection point at the same time as the 1.5T MRI in T2-weighted sequences (Graph 4).

## 5 Discussion

Many mesenchymal stem cell delivery methods have been developed for human and animal subjects, e.g., intra-articular injection, intramuscular injection, intravenous injection and 2- or 3-dimensional scaffold transplantation [3, 12]. Outcomes at follow-up have been measured in many ways. One outcome evaluation technique is histological assessment after treatment [22, 28, 29]. However, a problem with histopathological assessment in human subjects is that the surgeons or interventionists cannot perform a secondary invasive procedure unless there is a medical or surgical indication. Cell tracking with indirect visualization using cell tracers is one option for follow-up to confirm and determining the location of cell engraftment or cell deposits.

Iron sucrose is a cell marker that has been demonstrated to be safe for human use and human-MSCs [22, 28]. Iron sucrose does not affect either mesenchymal stem cell characteristics or cell viability. In addition, the cell differentiation potential of IS-labeled ASCs is not affected. Cells can be differentiated as adipocytes, osteocytes and chondrocytes using specific histological staining, similar to the report in a previous study [28].

This study is the first to demonstrate that IS-labeled ASCs are able to be detected using 1.5 and 3T MRI. Most published studies have reported using gadopentetate dimeglumine, ultrasmall paramagnetic iron oxides (USPIOs) and/or superparamagnetic iron oxides (SPIOs) for MRI cell tracking [30, 31, 32]; however, high cost and limited availability of that material limits their usefulness. Iron sucrose has been approved by the U.S.FDA for treatment of iron deficiency anemia and anemia resulting from chronic kidney diseases. Iron sucrose is not genotoxic, although cases of hypersensitivity have been reported [17].

T2∗-weighted sequences with 1.5 and 3T MRI is a more sensitive technique for detecting IS-labeled ASCs than T2-weighted sequences. T2∗-weighted sequences are presently used to detect iron deposits in tissue, e.g., paramagnetic deoxyhemoglobin, methemoglobin, and hemosiderin [29]. The minimal cell concentration that MRI can reliably detect using T2∗-weighted sequences is 1 × 10^5^ cells/ml/microtube. That has been demonstrated to be sufficient to detect MSCs engraftment in many recent clinical trials involving injection of MSCs at levels greater than 1 × 10^6^ cells/ml [7, 8, 10, 33, 34, 35, 36]. This study did not evaluate artifact effects from the iron particles; additional *in vivo* studies are needed to confirm and evaluate the quality of the images and any other effects related to iron particle artifacts.

Determination of the duration of detectability of IS-labeled ASCs was not one of the objectives of this study; however, a previous study using Lapine's intervertebral disc xenotransplantation model showed ASCs could be detected by histologic examination for at least 3 months and that the mean viability of IS-labeled MSCs was 99% at 1 month after transplantation which is not significantly different from non-iron sucrose-labeled MSCs (95%) [28]. In a human study, IS-labeled MSCs could be detected for at least 6–12 months in a transplanted intervertebral disc; extracellular iron particles were found in only one patient who had received a transplant 2.5 years previously; however, clinical outcomes, side effects and adverse reactions to IS-labeled ASCs and MSCs transplantation in human subjects were not reported in that study [26]. Currently, many aspects of IS-labeled ASCs and MSCs, e.g., the duration of cell detection by MRI, the longevity of cells after transfection in a human subject, the proliferation potential and clinical results of IS-labelled ASCs/MSCs transplantation, are largely unknown as there has not been much research on those topics. Further investigation is needed.

The main limitations of this study are the small sample size and the fact that this was designed as an *in vitro* study. This study needs to be shown to be reproducible with equipment from different manufactures, different MRI settings and different program analyses to compliment the 1.5 and 3T MRI scanners used this study and in an *in vivo* investigation.

This study used fetal bovine serum (FBS) in the cell culture medium. In patients, there would be a risk of FBS/media being contaminated with prions, viruses, mycoplasmas, or other unidentified zoonotic contaminants that could be transmitted to the host. FBS might cause an immune reaction during MSC therapy. Additional xenogeneic factors sometimes found in animal products, e.g., FBS or fetal calf serum [FCS], can induce hyperimmunogenic MSCs, causing rejection and subsequent failure of cell therapy as well as possible infection. The level of risk would be increased with multiple MSC transplants [39]. To avoid the risk of complications from FBS, an alternative approach to using cell medium in clinical practice is to use animal-free serum medium such as human-platelet lysate (HPL) medium [40] or commercial serum-free medium. Further experiments need to be conducted with both HPL and serum-free medium before their use in clinical practice.

## 6 Conclusions

Iron sucrose is a potential alternative cell tracer for use in regenerative medicine. IS-labeled ASCs is suitable for use in conventional MSCs or ASCs therapy in many situations e.g. orthopaedic-related disease/injury, to promote nerve healing, and for skin repair. It increases the accuracy of indirect and non-invasive evaluation in ASCs engraftment after treatment. At least 1 × 10^5^ cell/ml of IS-labeled ASCs can detected by 1.5 and 3T MRI with a T2∗-weighted sequence, indicating that iron sucrose is suitable for both further animal and human clinical studies as a viable alternative to superparamagnetic iron oxide (SPIOs). This technique could be useful in clinical practice and in stem-cell therapy clinics as part of patient follow-up procedures.

## Declarations

### Author contribution statement

Paween Tangchitphisut: Conceived and designed the experiments; Performed the experiments; Analyzed and interpreted the data; Contributed reagents, materials, analysis tools or data; Wrote the paper.

Narongrit Srikaew: Performed the experiments; Contributed reagents, materials, analysis tools or data.

Sith Phongkitkarun, Suphaneewan Jaovisidha: Contributed reagents, materials, analysis tools or data.

Tulyapruek Tawonsawatruk: Conceived and designed the experiments; Wrote the paper.

### Funding statement

This work was supported by a grant from 10.13039/501100004396Thailand Research Fund and the Faculty of Medicine, Ramathibodi Hospital, Mahidol University, Thailand.

### Competing interest statement

The authors declare no conflict of interest.

### Additional information

No additional information is available for this paper.
